# City Level of Income and Urbanization and Availability of Food Stores and Food Service Places in China

**DOI:** 10.1371/journal.pone.0148745

**Published:** 2016-03-03

**Authors:** Chunxiao Liao, Yayun Tan, Chaoqun Wu, Shengfeng Wang, Canqing Yu, Weihua Cao, Wenjing Gao, Jun Lv, Liming Li

**Affiliations:** Department of Epidemiology and Biostatistics, School of Public Health, Peking University, Beijing, China; Morehouse School of Medicine, UNITED STATES

## Abstract

**Objective:**

The contribution of unhealthy dietary patterns to the epidemic of obesity has been well recognized. Differences in availability of foods may have an important influence on individual eating behaviors and health disparities. This study examined the availability of food stores and food service places by city characteristics on city level of income and urbanization.

**Methods:**

The cross-sectional survey was comprised of two parts: (1) an on-site observation to measure availability of food stores and food service places in 12 cities of China; (2) an in-store survey to determine the presence of fresh/frozen vegetables or fruits in all food stores. Trained investigators walked all the streets/roads within study tracts to identify all the food outlets. An observational survey questionnaire was used in all food stores to determine the presence of fresh/frozen vegetables or fruits. Urbanization index was determined for each city using a principal components factor analysis. City level of income and urbanization and numbers of each type of food stores and food service places were examined using negative binomial regression models.

**Results:**

Large-sized supermarkets and specialty retailers had higher number of fresh/frozen vegetables or fruits sold compared to small/medium-sized markets. High-income versus low-income, high urbanized versus low urbanized areas had significantly more large-sized supermarkets and fewer small/medium-sized markets. In terms of restaurants, high urbanized cities had more western fast food restaurants and no statistically significant difference in the relative availability of any type of restaurants was found between high- and low-income areas.

**Conclusions:**

The findings suggested food environment disparities did exist in different cities of China.

## Introduction

The world’s obesity epidemic has risen threefold or more since the 1980s [1]. In parallel with the rising obesity epidemic, data based on nationwide surveys of food consumption patterns and household expenditures showed a marked upward trend in total energy intake derived from processed and pre-packaged foods for home use and meals away from home [2–3]. For example, 82% of US adults ate away from home at least once weekly[4] and the percentage of energy intake from away foods has increased to 32%[5]. China has also seen significant increases in prevalence of obesity and overweight over the past decades[6], and the food expenditures spent on eating away from home have risen threefold or more in city residents since 1991[3]. The prevalence of these unhealthy dietary behaviors is associated with higher intake of daily energy and has been related to the rising obesity epidemic [7–8].

Dietary behavior is complex, as it is determined by multiple levels of influences including individual, social and environmental levels [9–10]. Recently there has been a growing interest in the role of environment in promoting or hindering healthy eating. Emerging evidence suggested that characteristics of local residential environments significantly affected the availability of health food and dietary behaviors [11]. Large supermarket and retail grocery store chains as compared to small non-chain stores have been shown to be more likely to stock healthier foods at a lower cost [12–14]. People living in neighborhoods with more supermarkets and fewer convenience stores tended to have higher intake of fruits and vegetables [15–16] and lower prevalence of obesity [17]. Nevertheless, the most recent natural experiments [18] which showed moderate association of new supermarkets with residents’ perceptions of food accessibility but little with dietary change or body mass index (BMI) highlight the gap between perception and action among community residents. With respect to food service place, studies reported that fast food consumption was related to fast food availability [19] whereas no consistent evidence was found between food outlets near homes and dietary intake or BMI [20] which may suggest that eating out often happens outside the residential neighborhood and definitions of the neighborhood food environment should be in line with individual travel patterns [21].

During the period of 1993 to 2009, the prevalence of obesity increased from 4.0% to 10.7% in Chinese adults [6]. The obesity rates were shown to be significantly higher among high income groups in comparison to low income groups [22]. Evidence also showed that obesity prevalence was associated with increased degrees of urbanization [23]. Exploring the relationship between different local area food outlets availability and city developmental characteristics may help to understand the uneven distribution of obesity rates. Studies from western countries have investigated disparities in the food environment across socio-demographically varied geographical areas [24–25], but results of studies were mixed. While some studies conducted in Canada [26], New Zealand [27] and US [17] suggested that socioeconomically disadvantaged neighborhoods tended to have fewer supermarkets or more fast food restaurants. Other studies in major cities in the Denmark [28] and Australia [29] found low-income neighborhoods to have equal if not better access to supermarkets than more affluent ones.

China is undergoing rapid changes in lifestyle and dietary behavior among city residents [30]. Yet to date, it is not well known about the characteristics of food environment for cities at different stages of socio-economic development in China. It is the base on which a relevant and appropriate strategy on diet and health applicable to local needs stands. We conducted store audits in 12 cities of China from July to September 2012. This article aimed to describe the built nutritional environment in terms of types and numbers of food stores and food service places in relation to city level of income and urbanization in China.

## Methods

### Study setting and sample

**Fig 1:**
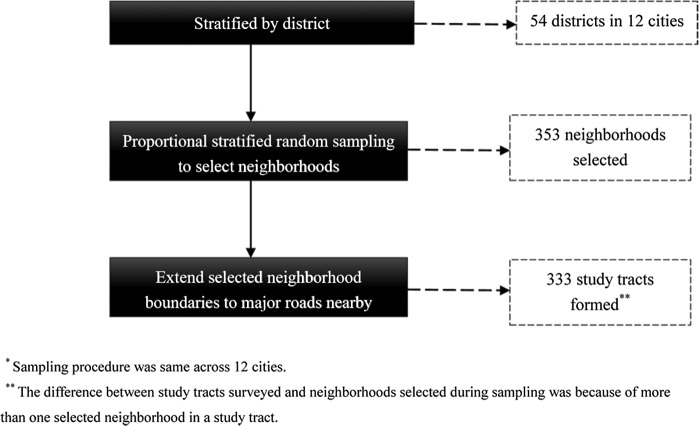
The sampling diagram of each city.

This study was conducted in 12 cities of China, including Beijing, Tianjin, Shanghai, Qingdao, Hangzhou, Shaoxing, Suzhou, Nantong, Zhenjiang, Chengdu, Xining and Harbin. In each city, neighborhoods were selected using a proportional stratified random sampling design on administrative district in central districts of the city. A typical neighborhood in most urban areas of China usually shows a shape of square or rectangle with 0.2 to 0.5 km^2^ in area. In this study, we extended sampled neighborhoods out from each side of the neighborhood boundaries to main road nearby to form a study area with 0.5 to 2.0 km^2^ in area. These extended study areas were regarded as our study tracts which represented availability to resources relative to the street network and potentially reflects routes of travel [31]. At last, 333 study tracts containing 948 neighborhoods covering an area of 224.68 km^2^ were surveyed from 54 central districts of the 12 cities (see Fig 1 for the sampling procedure). Sampling weights were calculated for each study tract as the inverse of the proportion of the study tracts in the district that were sampled.

### Measurement of Food Environment

Food stores and food service places were identified by systematically walking all the streets/roads within the study tracts by investigators. All the investigators were well trained through training seminars and pre-surveys. Global Positioning System data were collected in front of each location using a GPS. Three overall categories of food stores were identified: (1) large-sized supermarkets, defined as independent and chain supermarkets with an area of more than 6000 m^2^; (2) small/medium-sized markets, defined as variety and convenience stores and food stores attached to gasoline filling stations (e.g. convenience stores, corner stores) with an area of no more than 6000 m^2^; and (3) specialty retailers, defined as markets selling meat&fish or fruit&vegetable. Food service places were categorized as: (1) western fast food restaurants, selling western convenience food such as hamburg, pizza, pasta; (2) Chinese fast food restaurants, selling Chinese convenience and quick preparation food, usually in a single serve portion, such as Chinese combo, fried rice, noodels; (3) small-sized Chinese full-service restaurants (server brings food to table), defined as restaurants which provide variety dishes to order and accommodate no more than 75 sittings; (4) medium/large-sized Chinese full-service restaurants (server brings food to table), defined as restaurants which provide variety dishes and accommodate more than 75 sittings; and (5) tea & juice bars, defined as places mainly providing coffee, tea or juice. The primary outcome was the availability of food outlets measured as the numbers of food stores and food service places per 10000 population.

In this study, we classified the food stores into large-sized supermarkets, small/medium-sized markets and specialty retailers in relevance to different kinds of foods they provided. According to GB/T18106-2004 “Classification of retailing forms”, we choose the cutoff point as 6000m^2^ to define small/medium or large sized markets. In terms of food service places, fast food restaurants were distinguished from full-service restaurants due to different food types and nutritional value. We further classified full-service restaurants by size in that small-sized full-service restaurants differed from medium/large-sized full-service restaurants on the features of consumer groups, food prices, and food quality and we selected 75 sittings to define the size of restaurants according to “Administrative Measures for the Licensing of Catering Services” which was put in force in 2010 by the Minister of Health of the People’s Republic of China.

Quality control was conducted after the investigation by two senior trainers to estimate inter-investigator reliability. 44 study tracts covering 10% of all the food outlets were randomly selected as the test sample. The percentage of agreement among the two investigators was 94.7 indicating excellent reliability.

### In-store survey

An observational survey questionnaire was used in all food stores to determine the presence of the diversified food items (liquor, sugar-sweetened beverages, fresh/frozen vegetables or fruits). We simply looked for the presence of the type of the above items and aimed to identify the food stores selling fresh/frozen vegetables or fruits. Canned and dried fruits and vegetables were not considered in this study because these processed products tend to have additives to make them taste better including unhealthy amounts of extra salt and sugar.

### City socioeconomic and demographic data

City level of income was defined by per capita disposable income. City urbanization level was defined by an urbanization index which was derived from eight measures: per capita GDP, per capita consumption expenditure, per capita disposable income, number of taxies, number of buses, number of universities, urban road area, and resident population size. All data were extracted from “China Statistical Yearbook” (China National Bureau of Statistics, 2012). The area of each study tract was measured using Google Earth (Google Earth Pro 6.1.0.5001). The population size of each neighborhood was collected from local official statistics in 2011.

### Statistical analyses

All analyses were performed using STATA version 12.0 (Stata Corporation, College Station, TX); p < 0.05 was considered statistically significant.

Tertiles of per capita disposable income were used to group city level of income into low (<26600RMB), middle (≥26600RMB and <32900RMB), and high (≥32900RMB). Principal components factor analysis with varimax rotation was performed to reduce the eight measures to two factors which explained 92.1% of total variance. City urbanization index was generated for each city from the two factor scores using the percentage of variance each factor contribute as weight to calculate the total score. Tertiles of urbanization index were used to define low, medium, and high urbanized cities.

Our analyses proceeded in two steps. At first, descriptive analysis was used to assess the city characteristics and basic information of study tracts and the number of food outlets by city level of income and urbanization. Then we conducted in-store survey analysis to describe the percent of three types of food stores selling fresh/frozen vegetables or fruits. Second, multivariate regression models were used to evaluate the degree to which city level of income and urbanization influenced the availability of food outlets. Negative binomial regression models controlling for differences in study tract area and population size, with a sandwich estimator to correct the standard errors for city clustering and a sampling weight to adjust for varying probabilities of selection were estimated for each of our primary outcome variables: (1) number of each type of food stores per 10000 population, (2) number of each type of food service places per 10000 population. This analytic approach is appropriate for modelling count variables (e.g. the number of food stores per 10000 population), usually for over-dispersed variables.

## Results

Table 1 details the descriptive statistics for eight measures of city urbanization and the group results by city income and urbanization as well as the basic study tracts information of the 12 cities. This study included a total of 14126 food stores and 21497 food service places within 333 study tracts. Small/medium-sized markets were the most common type of food store, accounting for 68.9% of all food stores, followed by specialty retailers (29.1%) and large-sized stores (2.0%). In terms of food service places, small-sized Chinses full-service restaurants alone accounted for 66.4% of all food service places. Full details are described in S1 Table.

**Table 1:** City characteristics and basic information of study areas in the 12 cities of China.

	Beijing	Tianjin	Shanghai	Qingdao	Suzhou	Zhenjiang	Nantong	Hangzhou	Shaoxing	Chengdu	Xining	Harbin	Total
City characteristics													
Per capita disposable income (10000 RMB)	3.29	2.69	3.62	2.86	3.46	2.66	2.51	3.24	3.33	2.30	1.58	2.00	-
City income level	high	middle	high	middle	high	middle	low	middle	high	low	low	low	-
Per capita consumption expenditure (10000RMB)	2.20	1.84	2.51	1.93	2.23	1.55	1.56	2.14	2.04	1.67	1.06	1.62	-
Per-capita GDP (10000RMB)	8.17	8.52	8.26	7.55	10.21	7.40	5.60	10.14	7.58	4.94	3.47	4.27	-
Resident population(10000 population)	2019	1355	2347	880	1052	313	729	874	493	1407	121	1064	-
Number of universities	89	55	66	22	20	5	6	37	7	37	9	48	-
Number of taxies	66646	31940	50438	9683	4003	1253	1464	10048	917	15903	5516	15435	-
Number of buses	21628	7686	16589	5419	3665	1091	1135	7543	687	7188	1735	5395	-
Urban roads area(m^2^)	9164	10492	9481	6605	6089	1912	3011	4900	1283	6715	805	4114	-
Urbanization index	1.288	0.448	1.236	-0.057	0.178	-0.668	-0.640	0.210	-0.402	-0.097	-1.166	-0.329	-
City urbanization level	high	high	high	middle	middle	low	low	high	low	middle	low	middle	-
Basic information of study areas													
Number of administrative district involved in this study	6	6	9	4	6	2	2	6	1	5	4	3	54
Number of primary unit of survey	31	29	37	24	37	22	30	26	20	29	22	26	333
Study area (km^2^)	22.7	18.8	23.8	16.7	25.1	13.7	19.9	20.0	14.4	27.9	10.9	10.8	224.7
Population in study areas (10000 population)	45.0	51.7	122.7	27.8	32.4	10.0	10.6	29.7	30.8	46.1	12.2	30.6	449.5

In-store survey was successfully conducted in 11945 food stores. There were 8798 food stores in which no fresh/frozen vegetables or fruits was sold, accounting for 73.7% of all food stores. The percentage of food stores selling fresh/frozen vegetables or fruits was only 7.1% for small/medium-sized markets, compared with 64.6% for large-sized supermarkets and 92.7% for specialty retailers.

Table 2 shows the numbers of each type of food stores and food service places per 10000 population by groups of city level of income and urbanization. On average there were 82.3 food stores including 2.1 supermarkets, 57.0 small/medium-sized markets and 23.2 specialty retailers and a total number of 111.0 food service places per 10000 population in each study tracts. Densities of outlets varied by city level of income and urbanization. High-income cities tended to have lower densities of food stores and food service places by population. By city urbanization level, medium urbanized cites tended to have the highest densities of food stores and food service places by population.

**Table 2:** Numbers of food stores and food service places per 10000 population by city level of income and urbanization, mean (SE)^*^.

	Total	By per capita disposable income			By urbanization index		
	N = 333	Low	Middle	High	Low	Medium	High
		(N = 107)	(N = 101)	(N = 125)	(N = 94)	(N = 116)	(N = 123)
Food stores							
Large-sized supermarkets	2.1 (0.25)	1.6 (0.25)	2.1 (0.45)	2.2 (0.40)	1.6 (0.32)	2.2(0.54)	2.1(0.32)
Small/medium-sized markets	57.0 (11.70)	91.4 (14.00)	73.8 (24.92)	37.0 (6.91)	81.5 (12.07)	90.2 (13.61)	40.9 (10.91)
Specialty retailers	23.2 (5.26)	37.6 (14.35)	26.1 (10.41)	16.8 (3.95)	26.7 (5.73)	39.7 (12.00)	16.6 (4.39)
Total	82.3 (16.73)	130.6 (28.43)	101.9 (35.41)	56.0 (11.18)	109.9 (17.36)	132.0 (25.26)	59.5 (15.27)
Food service places							
Western fast food	3.0 (0.59)	2.1 (1.18)	2.9 (0.52)	3.4 (0.91)	1.7 (0.46)	2.5 (0.94)	3.4(0.78)
Chinese fast food	5.1 (1.41)	4.5 (1.55)	9.4 (2.90)	3.4 (1.07)	7.1 (1.06)	7.0 (2.61)	4.2 (1.65)
Medium/large-sized Chinese full-service	17.9 (2.39)	22.5 (4.59)	16.5 (5.69)	16.9 (2.77)	15.9 (1.95)	21.2 (3.85)	16.9 (3.22)
Small-sized Chinese full-service	75.7 (15.68)	121.5 (36.67)	97.4 (29.53)	49.3 (4.92)	88.3 (16.23)	129.2 (32.20)	54.0 (10.54)
Tea & juice bars	9.3 (2.20)	15.7 (5.90)	10.1 (4.83)	6.7 (0.30)	8.3(2.00)	14.7 (5.14)	7.5 (2.03)
Total	111.0 (19.20)	166.2 (39.62)	136.2 (39.90)	79.6 (6.40)	121.3 (17.08)	174.6 (33.84)	86.0 (15.79)

*SE adjusted for city clustering.

The results from negative binomial regression analyses on the availability of food stores are presented in Table 3. Large-sized supermarkets were significantly more common in high-income cities with small/medium-sized markets more common in low-income cities. The number of specialty retailers was higher in low-income cities but the result was without significance. High-income cities had 1.84 times the number of large-sized supermarkets and less than half (49%) of small/medium-sized markets compared to low-income cities. Examining food stores availability by city urbanization level, there was approximately 2 times as many large-sized supermarkets in high urbanized cities as in low urbanized cities, but small/medium-sized markets were significantly much less (only 69% as many) in high urbanized cities than low urbanized cities.

**Table 3:** Models of the relationship between the numbers of each type of food stores and city level of income and urbanization^*^.

	Large-sized supermarkets			Small/medium-sized markets			Specialty retailers		
	IRR	P	95%CI	IRR	P	95%CI	IRR	P	95%CI
Income level (reference group: low)									
Middle	1.35	0.160	(0.89,2.07)	0.72	0.329	(0.38,1.38)	0.66	0.383	(0.26,1.69)
High	1.84	0.046	(1.01,3.35)	0.51	0.022	(0.29,0.91)	0.55	0.196	(0.22,1.37)
Urbanization level (reference group: low)									
Medium	1.40	0.207	(0.83,2.39)	1.26	0.223	(0.87,1.82)	1.57	0.171	(0.82,2.99)
High	1.95	0.005	(1.08,3.55)	0.69	0.044	(0.39,1.23)	0.83	0.604	(0.42,1.66)

*IRR, incidence-rate ratio.

The population size and area of study areas were adjusted for each model.

Turning to the relative availability of food service places, the results based on negative binomial analyses are showed in Table 4. By city level of income, there were marginally more western fast food restaurants (p = 0.055) and fewer small-sized Chinese full service restaurants (p = 0.057) available in high-income cities. Differences in food service places availability were significant by city urbanization level. Western fast food restaurants and medium/large-sized Chinese full service restaurants were more prevalent in high urbanized cities which had 2.89 times the number of western fast food restaurants and 1.46 times the number of medium/large-sized Chinese full service restaurants compared to their low urbanized counterparts.

**Table 4:** Models of the relationship between the numbers of each type of food service places and city level of income and urbanization^*^.

	Western fast food			Chinese fast food			Medium/large-sized Chinese full-service			Small-sized Chinese full-service			Tea & juice bars		
	IRR	P	95%CI	IRR	P	95%CI	IRR	P	95%CI	IRR	P	95%CI	IRR	P	95%CI
Income level (reference group: low)															
Middle	1.51	0.350	(0.64,3.59)	1.88	0.102	(0.88,3.99)	0.63	0.175	(0.32,1.23)	0.74	0.459	(0.33,1.64)	0.55	0.328	(0.17,1.81)
High	2.13	0.055	(0.98,4.62)	0.91	0.809	(0.44,1.88)	0.90	0.501	(0.59,1.39)	0.50	0.057	(0.25,1.02)	0.51	0.103	(0.23,1.14)
Urbanization level (reference group: low)															
Medium	1.70	0.134	(0.85,3.41)	1.01	0.977	(0.53,1.92)	1.55	0.051	(1.00,2.42)	1.61	0.094	(0.92,2.80)	2.09	0.110	(0.85,5.17)
High	2.89	<0.001	(1.60,5.21)	0.80	0.539	(0.39,1.63)	1.46	0.037	(1.02,2.10)	0.82	0.415	(0.52,1.31)	1.23	0.316	(0.65,2.31)

^*^ IRR, incidence-rate ratio.

The population size and area of study areas were adjusted for each model.

## Discussion

This study provided a comprehensive view of differences in the types of food stores and food service places available by city level of income and urbanization in 12 cities across China. Overall, we found evidence of systematic differences in the location of food outlets by city characteristics. High-income and high urbanized cities tended to have more large-sized supermarkets and fewer small/medium-sized markets. As to food service places, western fast food restaurants and medium/large-sized Chinese full service restaurants were more likely to be located in high urbanized cities indicating urbanicity outweigh income level as the determination of the distribution of restaurants.

### Food stores availability and Policy implications

The results of availability of large-sized stores and small/medium-sized stores by city income level in this study were consistent with previous studies in USA [32–35]. Our results showed that there were two times more available large-sized supermarkets in high-income areas and on the other hand, low-income areas were found to have greater numbers of available small/medium-sized markets. Similarly, we found that high urbanized cities had more available large-sized supermarkets and few small/medium-sized markets compared to low urbanized cites.

The lack of availability of large-sized supermarkets in low-income and low urbanized areas was of particular concern given our previous results of the fresh/frozen vegetables or fruits availability by different types of food stores which showed 64.6% of large-sized supermarkets and 92.7% of specialty retailers versus a mere 7.1% of small/medium-sized markets provided fresh/frozen vegetables or fruits. Our results supported previous studies that have reported that residents living in low-income and less urban areas face more unhealthy food resources in large part as a result of a lack of supermarket availability [33,35].

Policies in western countries to increase access to healthy food in poor and less urban areas mainly focused on attracting supermarkets to these economically disadvantaged areas [35]. However, there are important trade-offs between large-sized supermarkets (which often require large available land). Our results showed that instead of large-sized stores, specialty retailers could also provide healthy foods like fresh vegetables and fruits, besides, farmers markets in terms of easy access and consequences for neighborhood street life (including social interactions between neighborhoods) may have healthful consequences [32] and could improve the food environment of disadvantaged cities. However, this should not be interpreted as a proof to encourage the negative effect of the establishment of large-sized supermarkets in disadvantaged cities. In the economic context of China, there exist barriers for supermarket investment in low-income communities concerning its profitability, location and customer demands. Access to fresh, quality, and affordable food is a crucial community, health, and quality of life concern. Each of the players capable of helping fill the gap-community groups, the public sector, and the food retail industry has an important role in pursuing this goal. Considering the many different factors between high-income and low-income areas and consumers, multiple tactics involving input from governmental, investors and community groups will be required for interventions to be successful.

### Food service places availability and Policy implications

Previous study findings in USA [17, 36], England [37] and Australia [38] showed low income areas had more fast food restaurants. Fast food contains high fat and energy [39] and is the cheapest and most available option for low income individuals [40] in western countries. However, in China, our result showed the trend was in opposite direction that western fast food restaurants were more available in higher-income and higher urbanized cities. Because of rapid income and economic growth and globalization, western fast food is favored by more and more people especially among children and adolescents in urban areas of China [41]. Thus, China offers a nice experiment to further test whether “fast food” availability has an adverse impact on weight health in general. In this study, we also found that there were more medium/large-sized full service restaurants in higher urbanized cities. Studies have proved that average daily caloric intake from full-service restaurants was higher compared with food made at home [42]. As such, the prevalence of western fast food restaurants and medium/large-sized full service restaurants may contribute to the unhealthy eating patterns in residents living in high urbanized cities.

According to food labeling regulations in China, restaurant dishes are not required to post nutrition information so that no information is available on the energy contents of these restaurant foods. In order to allow consumers to make informed choices about how much calories they consume, a national requirement for accurate calorie and nutrition labeling in all restaurants is needed.

### Strengths and Limitations

This study confirmed that food options differed across socio-demographically varied geographical areas. Rigorously designed and sampling strategy ensured enough variations on food environmental features and representativeness of the study sample.

In further addressing study limitations, first, because sufficient data were not available at neighborhood-level or district-level in Chinese macroeconomic data, this study was designed to describe the characteristics and availability of food outlets and healthy foods in city level. Another limitation existed in this study was the classification of food stores and food service places. Types of food stores and food service places could be classified by Standard Industry Classification (SIC) codes based on commercial database [33] in similar studies conducted in western countries. Because of lacking available national commercial data sources for all registered businesses in China, we had to rely on the on-site assessment by investigators. Accounting for the difficulty and subjectivity of this method, misclassification could not be avoided. In this study, however, the inter-investigator reliability was very high. At last, we merely showed food types and availability across regions, but not causal impact on obesity outcomes.

## Conclusions

In conclusion, we found differences in the availability of food stores and food service places across socio-demographically varied geographical areas. The results underscored the adverse implications of low availability of large-sized supermarkets in poor and less urban areas as well as high prevalence of western fast food restaurants in high urbanized areas. To improve access to healthy foods and allow consumers to make informed choices, public health officials may pursue the appropriate measures as mentioned above. In addition, description of area differences in the local food environment is an important step to understand disparities and social inequalities in health. Thus, future research will need to map a more accurate description of local food environment and then move beyond descriptive studies to investigations of the causality in the association between the availability of various food resources and residents’ diets and diet-related health outcomes.

## Supporting Information

S1 Dataset Data of this manuscript. (XLSX)

S1 Table Types and numbers of food stores and food service places identified in the 12 cities of China, n (%). (DOCX)
