# Supplementary material for: City Level of Income and Urbanization and Availability of Food Stores and Food Service Places in China
Source: PLoS One. 2016 Mar 3;11(3):e0148745. doi: 10.1371/journal.pone.0148745 (PMC4777357; doi:10.1371/journal.pone.0148745)
Supplement: S1 Table — (DOCX) [file pone.0148745.s002.docx]

**S1 Table. Types and numbers of food stores and food service places identified in the 12 cities of China, n (%)**

|  | Beijing | Tianjin | Shanghai | Qingdao | Suzhou | Zhenjiang | Nantong | Hangzhou | Shaoxing | Chengdu | Xining | Harbin | Total |
| --- | --- | --- | --- | --- | --- | --- | --- | --- | --- | --- | --- | --- | --- |
| **Food stores** | | | | | | | | | | | | | |
| Large-sized supermarkets | 34 | 20 | 53 | 10 | 21 | 19 | 16 | 31 | 22 | 25 | 15 | 12 | 278 |
|  | (2.5) | (2.3) | (3.0) | (0.8) | (1.7) | (3.1) | (2.0) | (2.9) | (2.8) | (1.3) | (1.5) | (0.8) | (2.0) |
| Small/medium-sized markets | 898 | 601 | 1208 | 908 | 813 | 409 | 544 | 687 | 524 | 1450 | 640 | 1050 | 9732 |
|  | (66.7) | (69.4) | (68.8) | (74.5) | (67.1) | (67.4) | (69.5) | (64.6) | (67.2) | (75.2) | (63.7) | (67.1) | (68.9) |
| Specialty retailers | 414 | 245 | 494 | 301 | 378 | 179 | 223 | 345 | 234 | 452 | 349 | 502 | 4116 |
|  | (30.8) | (28.3) | (28.1) | (24.7) | (31.2) | (29.5) | (28.5) | (32.5) | (30.0) | (23.5) | (34.8) | (32.1) | (29.1) |
| Total | 1346 | 866 | 1755 | 1219 | 1212 | 607 | 783 | 1063 | 780 | 1927 | 1004 | 1564 | 14126 |
| **Food service places** | | | | | | | | | | | | | |
| Western fast food | 89 | 60 | 88 | 39 | 46 | 24 | 20 | 35 | 32 | 40 | 20 | 36 | 529 |
|  | (3.3) | (4.2) | (3.6) | (2.4) | (2.6) | (2.6) | (1.9) | (1.9) | (2.7) | (1.4) | (1.5) | (1.6) | (2.5) |
| Chinese fast food | 133 | 88 | 56 | 111 | 137 | 51 | 70 | 148 | 117 | 46 | 107 | 78 | 1142 |
|  | (4.9) | (6.1) | (2.3) | (6.8) | (7.7) | (5.6) | (6.8) | (8.1) | (9.9) | (1.6) | (7.8) | (3.4) | (5.3) |
| Medium/large-sized Chinese full-service | 627 | 192 | 499 | 171 | 269 | 150 | 156 | 222 | 183 | 493 | 215 | 309 | 3486 |
|  | (23.3) | (13.4) | (20.4) | (10.5) | (15.2) | (16.5) | (15.0) | (12.1) | (15.5) | (17.2) | (15.6) | (13.4) | (16.2) |
| Small-sized Chinese full-service | 1598 | 1031 | 1574 | 1246 | 1126 | 576 | 696 | 1229 | 753 | 1682 | 985 | 1785 | 14281 |
|  | (59.3) | (72) | (64.3) | (76.2) | (63.5) | (63.3) | (67.1) | (66.9) | (63.8) | (58.5) | (71.5) | (77.6) | (66.4) |
| Tea & juice bars | 248 | 60 | 231 | 68 | 194 | 109 | 95 | 204 | 96 | 612 | 50 | 92 | 2059 |
|  | (9.2) | (4.2) | (9.4) | (4.2) | (10.9) | (12.0) | (9.2) | (11.1) | (8.1) | (21.3) | (3.6) | (4.0) | (9.6) |
| Total | 2695 | 1431 | 2448 | 1635 | 1772 | 910 | 1037 | 1838 | 1181 | 2873 | 1377 | 2300 | 21497 |
